# Spectroscopic characterization of two peroxyl radicals during the O_2_-oxidation of the methylthio radical

**DOI:** 10.1038/s42004-022-00637-z

**Published:** 2022-02-17

**Authors:** Zhuang Wu, Xin Shao, Bifeng Zhu, Lina Wang, Bo Lu, Tarek Trabelsi, Joseph S. Francisco, Xiaoqing Zeng

**Affiliations:** 1 Department of Chemistry, Shanghai Key Laboratory of Molecular Catalysts and Innovative Materials, Fudan University, Shanghai, 200433 China; 2 Department of Earth and Environment Science, University of Pennsylvania, Philadelphia, PA 19104-6243 USA

**Keywords:** Physical chemistry, Theoretical chemistry, Atmospheric chemistry, Infrared spectroscopy

## Abstract

The atmospheric oxidation of dimethyl sulfide (DMS) yields sulfuric acid and methane sulfonic acid (MSA), which are key precursors to new particles formed via homogeneous nucleation and further cluster growth in air masses. Comprehensive experimental and theoretical studies have suggested that the oxidation of DMS involves the formation of the methylthio radical (CH_3_S•), followed by its O_2_-oxidation reaction via the intermediacy of free radicals CH_3_SO_*x*_• (*x* = 1–4). Therefore, capturing these transient radicals and disclosing their reactivity are of vital importance in understanding the complex mechanism. Here, we report an optimized method for efficient gas-phase generation of CH_3_S• through flash pyrolysis of *S*-nitrosothiol CH_3_SNO, enabling us to study the O_2_-oxidation of CH_3_S• by combining matrix-isolation spectroscopy (IR and UV–vis) with quantum chemical computations at the CCSD(T)/aug-cc-pV(X + d)Z (X = D and T) level of theory. As the key intermediate for the initial oxidation of CH_3_S•, the peroxyl radical CH_3_SOO• forms by reacting with O_2_. Upon irradiation at 830 nm, CH_3_SOO• undergoes isomerization to the sulfonyl radical CH_3_SO_2_• in cryogenic matrixes (Ar, Ne, and N_2_), and the latter can further combine with O_2_ to yield another peroxyl radical CH_3_S(O)_2_OO• upon further irradiation at 440 nm. Subsequent UV-light irradiation (266 nm) causes dissociation of CH_3_S(O)_2_OO• to CH_3_SO_2_•, CH_2_O, SO_2_, and SO_3_. The IR spectroscopic identification of the two peroxyl radicals CH_3_SOO• and CH_3_S(O)_2_OO• is also supported by ^18^O- and ^13^C-isotope labeling experiments.

## Abstract (web summary)

The atmospheric oxidation of dimethyl sulfide affects the Earth’s climate, but the intricate pathways of this complex reaction are still not fully described. Here, the authors use an optimized flash pyrolysis method for gas-phase generation of the methylthio radical to study its O_2_-oxidation via matrix-isolation spectroscopy, and unambiguously identify two peroxyl radical intermediates.

## Introduction

Dimethyl sulfide (DMS, CH_3_SCH_3_) is the most abundant biogenic volatile organic sulfur compound (VOSC) that is produced through enzymatic lysis of dimethylsulfoniopropionate (DMSP) in the oceans^1–3^. On a global scale, marine DMS plays a key role in the organosulfur cycle with an estimated annual flux of about 30 teragrams of sulfur in the atmosphere^4,5^. The removal of DMS under marine atmospheric boundary layer (MABL) conditions involves biological consumption, sea-atmosphere exchange, and oxidation reactions. The atmospheric oxidation of DMS to condensable products contributes to the formation of secondary sulfate aerosols that affect Earth’s climate by scattering solar irradiation and simultaneously acting as cloud condensation nuclei (CCN)^6,7^. Therefore, the details about the oxidation mechanism of DMS are of vital importance in understanding the interplay between atmospheric chemistry and climate change^8^.

**Fig. 1:**
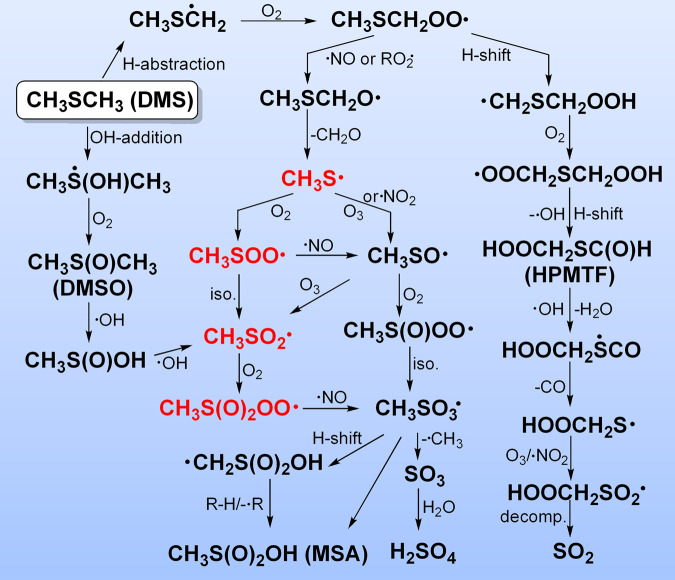
Reaction pathways for the atmospheric oxidation of dimethyl sulfide (DMS). The reaction pathways for the formation and subsequent O_2_-oxidation of methylthio radical (CH_3_S•) during the atmospheric oxidation of dimethyl sulfide (DMS). Radicals studied in this work are shown in red.

According to comprehensive smog chamber experiments and theoretical modeling^9–12^, the oxidation of DMS in the atmosphere is rather complex, which mainly invokes the formation of methylthio radical (CH_3_S•) through H-abstraction and subsequent radical-initiated decomposition upon reactions with •OH, •Cl, or •NO_3_ via the intermediacy of elusive radicals CH_3_SCH_2_•, CH_3_SCH_2_OO•, and CH_3_SCH_2_O• (Fig. 1). Then, the oxidation proceeds by further reactions of CH_3_S• with atmospherically relevant oxidants (e.g., O_2_, O_3_, and •NO_2_) to yield a number of transient sulfur-containing radicals including sulfinyl radical CH_3_SO•, sulfonyl radical CH_3_SO_2_•, and sulfonyloxyl radical CH_3_SO_3_•. Eventually, CH_3_SO_3_• can either dissociate (→ •CH_3_ + SO_3_) or undergo hydrogen abstraction to furnish sulfuric acid (SO_3_ + H_2_O → H_2_SO_4_) and methane sulfonic acid (CH_3_SO_3_H, MSA)^9^, respectively. Both acids are key precursors to new particles formed via homogeneous nucleation and subsequent cluster growth in air masses^13,14^. Recently, an alternative abstraction pathway for the •OH initiated oxidation of DMS to SO_2_ through the intramolecular H-shift in the common peroxyl radical intermediate CH_3_SCH_2_OO• (→ •CH_2_SCH_2_OOH) has been proposed (Fig. 1), in which the formation of the key stable intermediate hydroperoxymethyl thioformate (HPMTF, HOOCH_2_SCHO) has been confirmed experimentally^8,15–19^. On the other hand, the atmospheric oxidation of DMS can also proceed through the OH-addition pathway (Fig. 1), resulting the stepwise formation of additional VOSCs dimethyl sulfoxide (DMSO, CH_3_S(O)CH_3_), and methanesulfinic acid (CH_3_S(O)OH)^15–22^.

Among the O_2_-oxidation reactions of CH_3_S•, formation of three peroxyl radicals CH_3_SOO•, CH_3_S(O)OO•, and CH_3_S(O)_2_OO• has been also postulated^9–12^. Indeed, the sulfinylperoxy radical CH_3_S(O)OO• and its photodecomposition to •CH_3_ and SO_3_ via the intermediacy of CH_3_SO_3_• have been observed during the O_2_-oxidation of CH_3_SO•^23–25^. As the initial O_2_-oxidation product of CH_3_S•, CH_3_SOO• is less stable than CH_3_S(O)OO• due to a small S–OO bond dissociation energy (BDE) of ca. 10 kcal mol^−1 ^^26,27^, and it has been only tentatively identified among the photolytic O_2_-oxidation products of CH_3_SSCH_3_ based on the observation of two transient absorptions using step-scan IR spectroscopy^28^. As the formal O_2_-oxidation product of the sulfonyl radical CH_3_SO_2_•^29–32^, CH_3_S(O)_2_OO• remains yet unobserved, although reactions between CH_2_SO_2_• and O_2_ via the intermediacy of CH_3_S(O)_2_OO• have been proposed during the one-electron reduction of CH_3_S(O)_2_Cl in oxygenated solutions^33^ and also in the pulse radiolysis of an N_2_O–O_2_ saturated solution of NaOS(O)CH_3_^34^. According to the recent theoretical computations, CH_3_S(O)_2_OO• has higher stability than CH_3_SOO• and CH_3_S(O)OO• due to the highest BDE for the shortest S–OO bond^35^.

To unveil the mechanism for the oxidation of CH_3_S•, a practical method for efficient generation of this thiyl radical is desirable. Typically, thiyl radicals (RS•) can be generated through homolytic cleavage of the S–S or S–H bonds in disulfides (RS–SR) or thiols (RS–H) under photolysis or pyrolysis conditions^36^. However, the associated large BDEs (>60 kcal mol^−1^) in these compounds render the efficiency of thermal fragmentation relatively low in the absence of catalyst^37^. Hence, UV-laser photolysis of CH_3_SSCH_3_ and CH_3_SH has been frequently used in generating CH_3_S• in the gas phase^38–40^. Recently, the formation of CH_3_S• from the decomposition of CH_3_SSCH_3_ was observed on metal surfaces by using visible light irradiation^41,42^, in which the photo-induced plasmon serve as the catalyst.

Herein, we report an optimized method for facile generation of CH_3_S• by high-vacuum flash pyrolysis (HVFP) of *S*-nitrosothiol CH_3_SNO in the gas phase, which enables us to study the mechanism for the O_2_-oxidation reactions of CH_3_S• and the first-time unambiguous identification of the two important intermediates CH_3_SOO• and CH_3_S(O)_2_OO• that are critical to the validation of the DMS oxidation process occurring in the atmosphere (Fig. 1).

## Results and discussion

### Generation of CH_3_S•

**Fig. 2:**
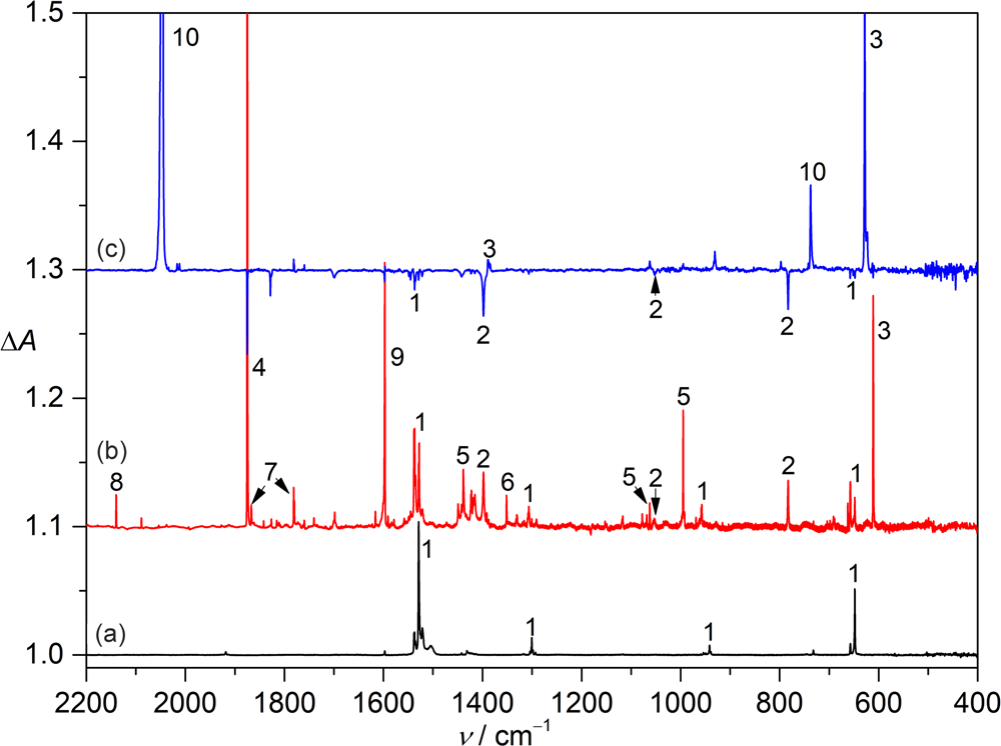
IR spectra showing the generation and photochemistry of CH_3_S• (2). **a** IR spectrum of CH_3_SNO in N_2_-matrix at 10 K. **b** IR spectrum of the high-vacuum flash pyrolysis (HVFP) products of CH_3_SNO in N_2_-matrix. **c** IR difference spectrum reflecting the change of the HVFP products upon UV-light irradiation (365 nm, 15 min). The IR bands for CH_3_SNO (**1**), CH_3_S• (**2**), •CH_3_ (**3**), •NO (**4**), H_2_CS (**5**), SO_2_ (**6**), N_2_O_2_ (**7**), CO (**8**), H_2_O (**9**), and N_2_S (**10**) are labeled.

*S*-nitrosothiols are endogenous sources of nitric oxide (•NO) in biological systems^43^ due to easy breakage of the S−N bonds with BDEs less than 30 kcal mol^−1 ^^44,45^. Particularly, the S−N bond energy in CH_3_SNO (**1**) is about 20 kcal mol^−1 ^^46^, implying facile fragmentation under pyrolysis conditions. A typical IR spectrum for the pyrolysis (400 °C) products of CH_3_SNO (Fig. 2a) isolated in N_2_-matrix at 10 K shows the formation of CH_3_S• (**2**, 1398.3, 1053.4, and 783.0 cm^−1^, Fig. 2b)^39^, •CH_3_ (**3**, 611.1 cm^−1^)^47^, and •NO (**4**, 1874.9 cm^−1^)^46^. Owing to the moderate BDEs for C–S (70 kcal mol^−1^) and C–H (49 kcal mol^−1^) bonds in **2**^38^, further increase of the pyrolysis temperature to ca. 650 °C leads to complete dissociation of **2** to **3** and H_2_CS (**5**, 1438.7, 1062.7, and 994.9 cm^−1^)^48^ through the elimination of sulfur and hydrogen atoms, respectively. When using ^13^C-labeled CH_3_SNO as the precursor, the isotopically labeled ^13^CH_3_S• can be generated, and noticeable ^12/13^C-isotopic shifts of 2.7, 6.2, and 4.8 cm^−1^ for the aforementioned three IR fundamental modes of **2** have been determined for the first time.

Upon irradiation with UV-light emitting diode (LED, 365 nm), the corresponding IR difference spectrum (Fig. 2c) reflecting the change of the matrix-isolated pyrolysis products of **1** shows primary depletion of **2** with concomitant formation of N_2_S (**10**, 2047.8 and 737.5 cm^−1^)^49^ and •CH_3_ (**3**, 628.1 cm^−1^). Therefore, the thiyl radical **2** acts as an effective sulfur atom transfer (SAT) reagent^50^ by reacting with the matrix material (N_2_) under the photolytic excitation at 365 nm. The identification of **10** is confirmed by the observation of large ^14/15^N-isotopic shifts of 67.8 and 13.1 cm^−1^ for the two bands in the experiment using ^15^N_2_ as the matrix material. The noticeable shift (Δ*ν* = 17.0 cm^−1^) of the IR band at 628.1 cm^−1^ for the newly formed **3** during the photolysis implies strong interactions with neighboring N_2_S in the same N_2_-matrix. The generation of CH_3_S• by pyrolysis of CH_3_SNO is reproducible when using Ar or Ne as carrying gas (Supplementary Fig. 1), and its photodecomposition to H_2_CS (**5**) was observed under similar UV-irradiation conditions (365 nm). It should be noted that the photoactivity of **2** coincides with the observed absorption at 375 nm for the radical in Ar-matrix (vide infra).

### O_2_-oxidation of CH_3_S•

**Fig. 3:**
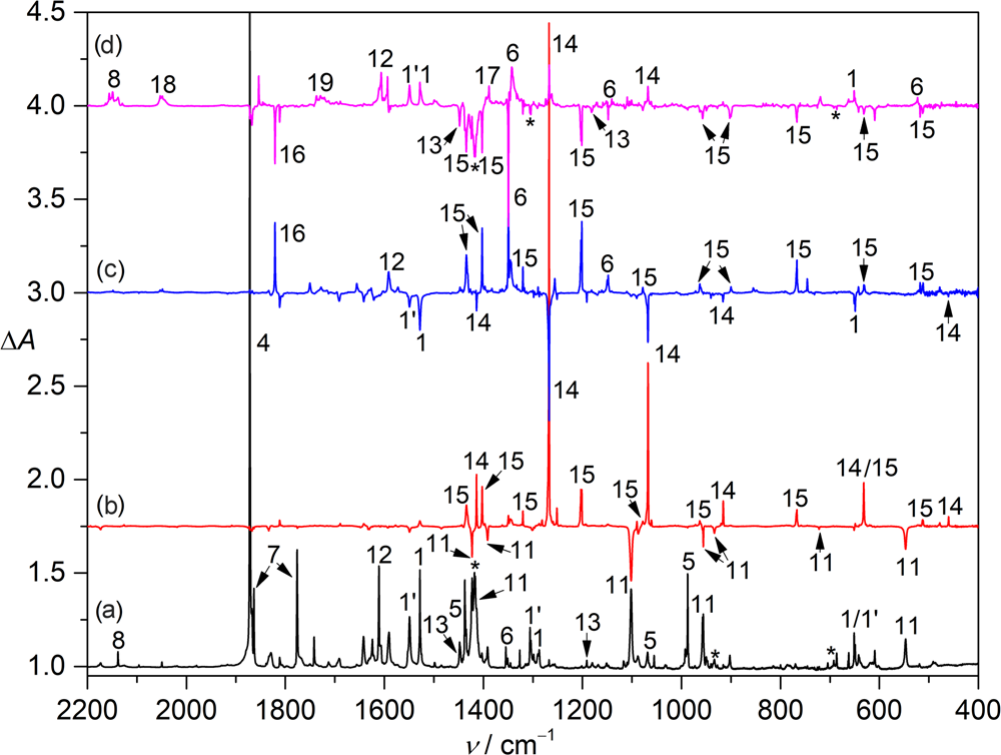
IR spectra showing the formation and photochemistry of CH_3_SOO• (11) and CH_3_S(O)_2_OO• (15). **a** IR spectrum of the HVFP products of CH_3_SNO/O_2_/Ar (1:50:1000) at 10 K. **b** IR difference spectrum reflecting the change of the HVFP products upon red-light irradiation (830 nm, 20 min). **c** IR difference spectrum reflecting the change of the HVFP products upon subsequent blue-light irradiation (440 nm, 40 min). **d** IR difference spectrum reflecting the change of the HVFP products upon further UV-laser irradiation (266 nm, 10 min). The IR bands for CH_3_SNO (*cis*: **1;** *trans*: **1′**), •NO (**4**), H_2_CS (**5**), SO_2_ (**6**), N_2_O_2_ (**7**), CO (**8**), CH_3_SOO• (**11**), NO_2_• (**12**), CH_3_OO• (**13**), CH_3_SO_2_• (**14**), CH_3_S(O)_2_OO• (**15**), CH_3_S•···•ON (**16**), SO_3_ (**17**), OCS (**18**), CH_2_O (**19**), and CH_3_SSCH_3_ (*) are labeled.

When the pyrolysis of CH_3_SNO (**1**) was performed in presence of oxygen (**1**/O_2_/Ar, 1:50:1000), the IR spectrum of the products at 10 K (Fig. 3a) shows complete disappearance of •CH_3_ and CH_3_S• by forming CH_3_OO• (**13**, 1447.8 and 1180.4 cm^−1^)^51^ and a new species (**11**) with strong IR bands at 1392.2 and 1102.2 cm^−1^ (Fig. 3a). These frequencies are close to the two transient absorptions at 1397 ± 1 and 1110 ± 3 cm^−1^ that were tentatively assigned to CH_3_SOO• in the previous gas-phase study on the photolytic (248 nm) O_2_-oxidation of CH_3_SSCH_3_^28^. In order to distinguish the IR bands for the most likely candidate (**11**), the matrix was subjected to a red-light LED irradiation (830 nm). The resulting IR difference spectrum shows exclusive depletion of **11** (Fig. 3b), and sulfonyl radical CH_3_SO_2_• (1413.9, 1274.2, 1074.5, 915.6, 631.3, and 460.2 cm^−1^, **14**)^29^ forms, indicating isomerization of **11** under the irradiation conditions. Similar photoisomerization has been found for PhSOO• (→ PhSO_2_•)^52^ and OSOO (→ SO_3_)^53^. The previously^28^ proposed secondary oxidation of **11** with O_2_ to form CH_3_SO• in the gas phase was not observed under the matrix-isolation conditions.

**Fig. 4:**
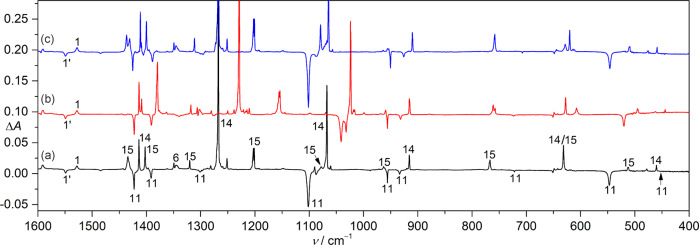
IR difference spectra for the isotope-labeling experiments. **a** Expaned IR difference spectra (1600–400 cm^−1^) for the photo-induced (830 nm) conversion of CH_3_SOO• (**11**) to CH_3_SO_2_• (**14**) and CH_3_S(O)_2_OO• (**15**). The spectra for the experiments with the ^18^O- and ^13^C-labeling samples are shown in **b** and **c**, respectively.

The selective conversion (**11** → **14**) allows unambiguous identification of all the remaining weaker IR fundamental bands for **11** (Table 1). The assignment is supported by the agreement with the CCSD(T)/aug-cc-pV(T + d)Z computed IR spectrum for **11** (Table 1) in a favorable *syn*-conformation between the CH_3_ moiety and the terminal oxygen atom with respect to the S–O bond. According to the ^18^O-isotope labeling experiment (Fig. 4), the strong band in CH_3_SOO• at 1102.2 cm^−1^ (calc. 1135.9 cm^−1^) is reasonably assigned to the O–O stretching mode (ν(OO)) due to a large ^16/18^O isotopic shift of 61.3 cm^−1^ (calc. 71.5 cm^−1^). In contrast, no shift occurs to this band in the ^13^C-isotope labeling experiment (Fig. 4). The frequency is close to the ν(OO) mode in other peroxyl radicals such as PhSOO• (1173 cm^−1^)^52^ and CH_3_S(O)OO• (*syn/anti*: 1100.3/1081.3 cm^−1^)^23^ with comparable ^16/18^O-isotopic shifts of 64 and 61.0/58.3 cm^−1^, respectively. The bands at 547.6 and 443.7 cm^−1^ correspond to the S–O stretching (calc. 571.1 cm^−1^) and SOO bending modes (calc. 406.0 cm^−1^) with large ^16/18^O-isotopic shifts of 27.0 (calc. 29.0 cm^−1^) and 15.3 cm^−1^ (calc. 10.7 cm^−1^) but small ^12/13^C-isotopic shifts of 0.8 (calc. 1.0 cm^−1^) and 0.5 cm^−1^ (calc. 0.3 cm^−1^), respectively. The C–S stretching mode locates at 722.5 cm^−1^ as it displays a large ^12/13^C-isotopic shift of 15.2 cm^−1^ (calc. 14.4 cm^−1^, Table 1), and it is close to the same mode in CH_3_SO_3_• (757.6 cm^−1^, Ar-matrix) and CH_3_S(O)OO• (*syn/anti*: 686.4/676.7 cm^−1^)^23^.

**Table 1:** Observed and calculated IR data (>400 cm^−1^) for CH_3_SOO•.

*ν*_obs._^a^			*ν*_cal._^b^		Δ*ν*(^16/18^O)^c^		Δ*ν*(^12/13^C)^c^		Assignment^d^
Ar-matrix	Ne-matrix	N_2_-matrix	CCSD(T)	M06-2X	obs.	cal.	obs.	cal.	
3012.9 (<1)	3012.5	3010.0	3158.4	3168.0 (<1)	n.o.^e^	<0.1	n.o.^e^	12.0	*ν*_1_, A”, ν_as_(CH_3_)
2995.9 (1)	2998.4	2998.8	3136.6	3147.3 (2)	<0.5	<0.1	11.5	10.7	*ν*_2_, A”, ν_as_(CH_3_)
2925.1 (2)	2932.2	2927.4	3044.9	3056.5 (1)	<0.5	<0.1	3.1	2.8	*ν*_3_, A’, ν_s_(CH_3_)
1422.8 (14)	1428.9	1423.7	1487.0	1477.0 (12)	<0.5	<0.1	−2.5	2.6	*ν*_4_, A’, δ(CH_3_)
1392.2 (13)	1396.4	1394.9	1450.1	1441.2 (9)	<0.5	<0.1	2.6	2.2	*ν*_5_, A”, δ(CH_3_)
1301.7 (6)	1307.2	1305.0	1346.4	1344.1 (1)	n.o.^e^	0.9	6.3	7.3	*ν*_6_, A’, δ(CH_3_)
1102.2 (100)	1107.8	1102.2	1135.9	1265.7 (31)	61.3	71.5	<0.5	0.2	*ν*_7_, A’, ν(OO)
956.4 (11)	960.0	957.9	987.1	973.9 (4)	<0.5	<0.1	5.5	5.5	*ν*_8_, A”, ω(CH_3_)
934.0 (9)	936.4	936.7	967.1	966.6 (5)	1.7	1.9	8.1	8.4	*ν*_9_, A’, ρ(CH_3_)
722.5 (1)	727.0	722.3	733.5	748.1 (1)	−1.4	0.1	15.2	15.3	*ν*_10_, A’, ν(CS)
547.6 (36)	549.4	555.0	571.1	621.4 (17)	27.0	29.0	0.8	1.0	*ν*_11_, A’, ν(SO)
443.7 (<1)	444.6	444.3	406.0	467.8 (<1)	15.3	14.4	0.5	0.3	*ν*_12_, A’, δ(SOO)

^a^Observed band positions for the most intense matrix sites and relative intensities (in parentheses) based on integrated band areas.

^b^Harmonic frequencies (>400 cm^−^^1^) and intensities (km mol^−^^1^, in parentheses) for the IR fundamentals calculated at the CCSD(T)/aug-cc-pV(T+d)Z and M06-2X/6-311++G(3df,3pd) levels of theory. Complete list of the IR data is given in Supplementary Table 1.

^c^M06-2X/6-311++G(3df,3pd) calculated and observed ^16/18^O- and ^12/13^C-isotopic shifts.

^d^Assignment of the vibration modes based on the vibrational displacement vectors.

^e^Not observed due to overlap or low intensity.

In addition to CH_3_SO_2_• (**14**), another species (**15**) with distinct IR bands at 1435.3, 1402.8, 1320.9, 1204.2, 1078.2, and 767.7 cm^−1^ also forms after the red-light irradiation of CH_3_SOO• (**11**) in the O_2_-doped Ar-matrix (Fig. 3b). Further irradiation of the same matrix with blue-light LED (440 nm) leads to specific conversion of **14** to **15** (Fig. 3c) together with the previously observed photodissociation of the two conformers of CH_3_SNO (*cis*: **1**; *trans*: **1’**) to the metastable caged radical pair CH_3_S•···•ON (**16**, 1820.9 cm^−1^) in the cryogenic matrix^46^. In the ^18^O-labeling experiment (Fig. 4), the two bands at 1435.3 and 1204.2 cm^−1^ shift to 1382.1 and 1156.5 cm^−1^, corresponding to isotopic shifts of 53.2 and 47.7 cm^−1^, respectively, whereas, only very small shifts occur to the two bands in the ^13^C-isotope labeling experiment. The frequencies are close to the two SO_2_ stretching modes (ν_as_(SO_2_) and ν_s_(SO_2_)) in methane sulfonic acid CH_3_SO_3_H (1403 and 1202 cm^−1^)^24^ and sulfonyl nitrene FS(O)_2_N (1426.4 and 1206.5 cm^−1^)^54^. In contrast, they are significantly higher than the ν_as_(SO_2_) and ν_s_(SO_2_) modes in **14** at 1274.2 and 1074.5 cm^−1^, for which the ^18^O-isotopic shifts are 38.9 and 44.1 cm^−1^, respectively. Additionally, the weak band at 1078.2 cm^−1^ displays a large ^18^O-isotopic shift of 59.6 cm^−1^. The frequency and associated isotopic shift are similar with the ν(OO) mode in CH_3_SOO• (1102.2 cm^−1^, Δν(^16/18^O) = 61.3 cm^−1^), strongly suggesting the assignment of this new species to the peroxyl radical CH_3_S(O)_2_OO• (**15**), which is formed from CH_3_SO_2_• (**14**) by further combination of molecular oxygen in the O_2_-doped Ar-matrix. The efficient isomerization of **11** to **14** is consistent with the theoretically predicted higher stability of the latter by a free energy difference (Δ*G*) of –65 kcal mol^−1^ (M06-2X/6-311++G(3df,3pd). In contrast, the subsequent conversion of **14** to **15** is a near thermodynamically neutral process with a calculated free energy change of –1.5 kcal mol^−1^.

The assignment of the IR bands for **15** is also supported by the agreement with the CCSD(T)/aug-cc-pV(D + d)Z computations (Table 2). For instance, the computed frequency for the ν(OO) mode in **15** is 1061.5 cm^−1^ (obs. 1078.2 cm^−1^), and the ν(SO) frequency at 621.2 cm^−1^ (Δ*ν*(^16/18^O) = 25.1 cm^−1^) matches the observation at 631.3 cm^−1^ (Δ*ν*(^16/18^O) = 24.0 cm^−1^). The experimentally observed ^16/18^O-isotopic shift of 53.2 cm^−1^ for the *ν*_as_(SO_2_) mode at 1435.3 cm^−1^ (cal. 1436.0 cm^−1^) agrees with the predicted ^16/18^O-shifts of 58.3 cm^−1^, and it mixes with the δ(CH_3_) mode at 1402.8 cm^−1^ (cal. 1367.9 cm^−1^) as evidenced by the ^16/18^O-isotopic shift of −7.5 cm^−1^ (cal. −14.9 cm^−1^). The distinguishment of the *ν*_as_(SO_2_) mode from the three δ(CH_3_) modes in the range of 1500–1350 cm^−1^ can be also acertained with their distinct ^12/13^C-isotopic shifts (Table 2). The generation of **11** and its photoisomerization to **14** with further oxidation to **15** is reproducible in N_2_- and Ne-matrixes (Supplementary Figs. 2 and 3).

**Table 2:** Observed and calculated IR data (>400 cm^−1^) for CH_3_S(O)_2_OO•.

*ν*_obs_^a^			*ν*_cal_^b^		Δ*ν*(^16/18^O)^c^		Δ*ν*(^12/13^C)^c^		Assignment^d^
Ar-matrix	Ne-matrix	N_2_-matrix	CCSD(T)	M06-2X	obs.	cal.	obs.	cal.	
n.o.^e^	3052.9	3053.0	3182.3	3193.8 (4)	n.o.^e^	<0.1	n.o.^e^	12.2	*ν*_1_, *ν*_as_(CH_3_)
3032.1 (3)	3036.5	3035.5	3167.7	3186.7 (5)	<0.5	<0.1	10.4	11.9	*ν*_2_, *ν*_as_(CH_3_)
2938.7 (1)	2958.0	2953.4	3054.2	3078.6 (2)	<0.5	<0.1	2.6	2.6	*ν*_3_, *ν*_s_(CH_3_)
1435.3 (98)	1439.7	1432.4	1436.0	1494.4 (191)	53.2	58.3	−2.2	<0.1	*ν*_4_, *ν*_as_(SO_2_)
1431.7 (<1)	n.o.^e^	n.o.^e^	1430.2	1459.4 (10)	n.o.	1.3	0.6	2.1	*ν*_5_, δ(CH_3_)
1402.8 (60)	1408.8	1403.3	1367.9	1449.2 (52)	−7.5	−14.9	2.5	2.0	*ν*_6_, δ(CH_3_)
1320.9 (18)	1324.4	1325.8	1317.2	1358.4 (34)	1.7	1.9	9.6	9.9	*ν*_7_, δ(CH_3_)
1204.2 (100)	1207.5	1203.1	1145.2	1255.6 (148)	47.7	49.9	0.5	0.1	*ν*_8_, *ν*_s_(SO_2_)
1078.2 (10)	1080.6	1079.7	1061.5	1237.5 (34)	59.6	70.6	<0.5	<0.1	ν_9_, *ν*(OO)
971.5 (2)	975.4	973.4	966.8	990.6 (6)	n.o.^e^	2.8	7.6	8.3	*ν*_10_, ω(CH_3_)
963.0 (18)	965.3	964.9	960.2	978.7 (33)	1.4	2.4	8.5	7.8	*ν*_11_, *ρ*(CH_3_)
767.7 (54)	772.7	770.9	757.6	802.9 (82)	7.5	8.8	8.9	9.0	*ν*_12_, *ν*(CS)
631.3 (12)	637.9	632.1	621.2	685.9 (64)	24.0	26.3	2.8	3.8	*ν*_13_, *ν*(SO)
513.1 (6)	514.9	513.6	488.9	531.4 (48)	17.6	18.5	3.0	2.3	*ν*_14_, δ(SO_2_)
479.4 (5)	480.9	481.9	461.8	496.3 (49)	16.0	16.9	2.7	2.7	*ν*_15_, δ(SOO)

^a^Observed band positions for the most intense matrix sites and relative intensities (in parentheses) based on integrated band areas.

^b^Harmonic IR frequencies (>400 cm^−^^1^) and intensities (km mol^−^^1^, in parentheses) for the IR fundamentals calculated at the CCSD(T)/aug-cc-pV(D + d)Z and M06-2X/6-311++G(3df,3pd) levels of theory. Complete list of the IR data is given in Supplementary Table 3.

^c^M06-2X/6-311++G(3df,3pd) calculated and observed ^16/18^O- and ^12/13^C-isotopic shifts.

^d^Assignment of the vibration modes based on the vibrational displacement vectors.

^e^Not observed due to overlap or low intensity.

In line with the computed lowest-energy vertical transition at about 220 nm for CH_3_S(O)_2_OO• (Supplementary Table 2), no change occurs to this peroxyl radical under visible-light irradiations. In contrast, it can be partly depleted by a 266 nm laser with unspecific decomposition (Fig. 3d) to SO_2_ (**6**), CO (**8**), CH_3_SO_2_• (**14**), SO_3_ (**17**), OCS (**18**), and CH_2_O (**19**). The formation of OCS in the oxidation of CH_3_S• is consistent with the previous discovery of the tropospheric oxidation of DMS as a potent source of OCS^55,56^, which serves as a key tracer for the global carbon cycle. The formation of SO_3_ indicates the possible involvement of CH_3_SO_3_• (CH_3_S(O)_2_OO• + O_2_ → CH_3_SO_3_• + O_3_), which can decompose (CH_3_SO_3_• → •CH_3_ + SO_3_) upon UV-light irradiation^23,24^. As a further step, photofragmentation of CH_3_OO• (**13**) to CO (**8**), CH_2_O (**19**), and CO_2_ occurs upon the laser irradiation.

### Electronic spectra

**Fig. 5:**
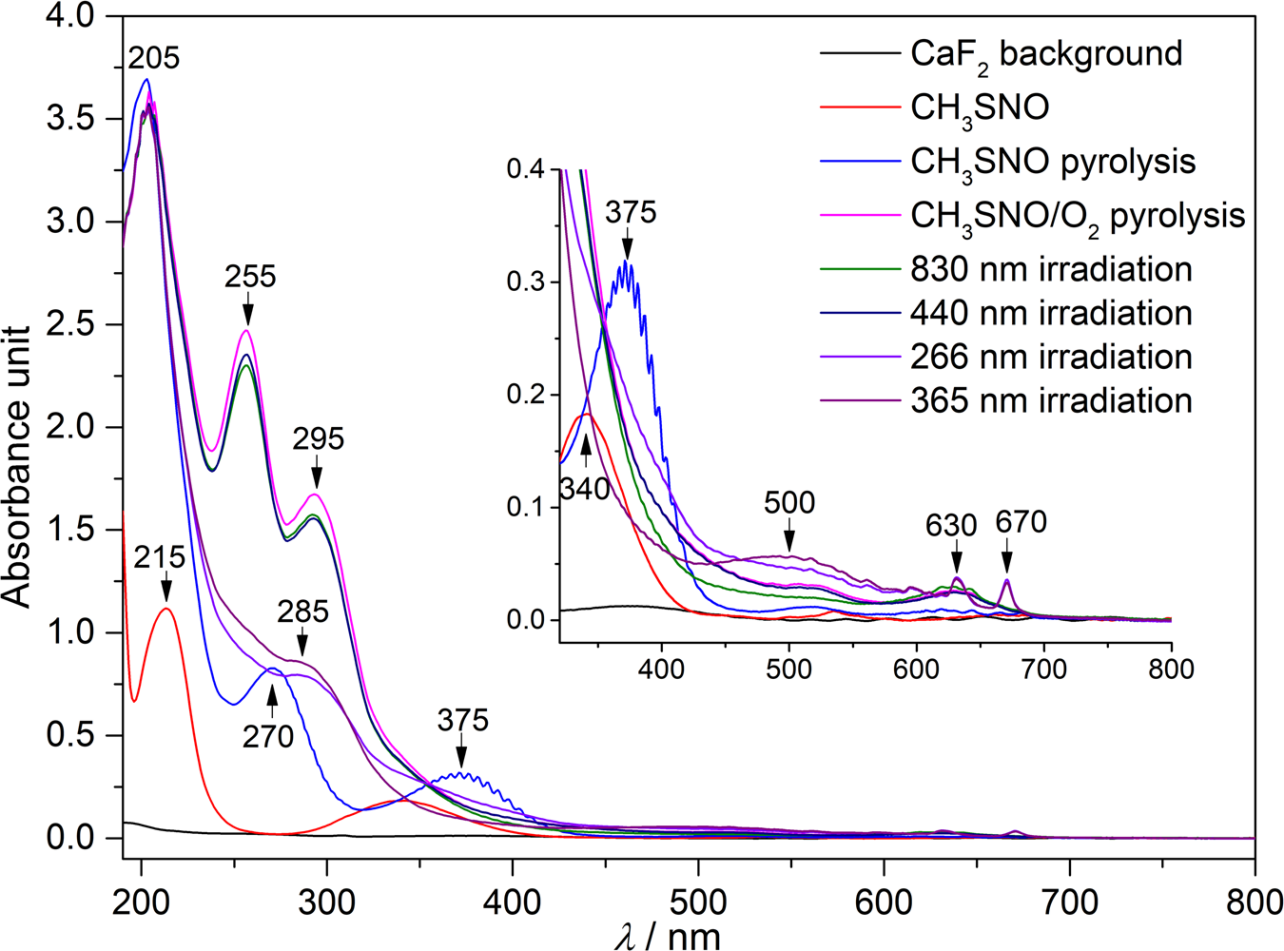
UV–vis spectra of CH_3_S• and its O_2_-oxidation products in Ar-matrix. UV–vis spectra of Ar-matrix isolated CH_3_SNO, HVFP products of CH_3_SNO, HVFP products of CH_3_SNO/O_2_, and the photolysis products of the HVFP products of CH_3_SNO/O_2_ at 10 K. Inset: the expanded spectra in the range of 800–320 nm.

The stepwise O_2_-oxidation reactions of CH_3_S• (**2**) via the intermediacy of peroxyl radicals were also followed with matrix-isolation UV–vis spectroscopy. Thanks to the efficient production of **2** in the gas phase, a full UV–vis absorption spectrum for this simplest organosulfur radical isolated in an Ar-matrix at 10 K has been obtained (Fig. 5). Note that only weak absorptions in the range of 220–200 nm were assigned to **2** in previous gas-phase studies^57–59^. In sharp contrast, **2** isolated in Ar-matrix displays two absorptions that completely differ from its precursor (**1**, 340 and 215 nm). The weak absorption band (*λ*_max_) of **2** at 375 nm exhibits pronounced vibrational fine structures with onset at ca. 450 nm. This assignment coincides with the previous MRCI computed energy of 373 nm for the Ã(^2^*A*_1_) ← *X̃* ^2^*E* transition^60^, and it also reasonably explains the aforementioned photochemistry of **2** by the irradiation at 365 nm (Fig. 2c). The second stronger band of **2** at 270 nm contains superimposed vibrational fine structures for the byproduct S_2_ that is generated via fragmentation of **2** (→ •CH_3_ + S) followed by immediate aggregation during the same deposition process. The assignment of the strongest band at 205 nm is unclear since other accompanied decomposition products (S_2_, •NO, and •CH_3_) of undecomposed CH_3_SNO in the same matrix also contain absorptions at around 200 nm.

In the UV–vis spectrum of the matrix-isolated HVFP products of CH_3_SNO/O_2_, the absorption bands for CH_3_S• (375 and 270 nm) disappear while two new overlapping bands occur at 295 and 255 nm with onset near 400 nm. Subsequent irradiation with 830 nm light causes partial depletion of the broad absorption, implying the contribution of the absorption from the highly photolabile peroxyl radical CH_3_SOO• (**11**). In the same time, absorptions for the products CH_3_SO_2_• (**14**) in the range of 330–400 nm^27,30^ and CH_3_S(O)_2_OO• (**15**) with predicted intense absorption at 221 nm should appear by referring to the corresponding IR spectrum (Fig. 3b). The byproduct CH_3_OO• (**13**)^61^ formed in the pyrolysis of CH_3_SNO/O_2_ also contributes to the broad band, and it remains unchanged during the successive visible light irradiations (830 and 440 nm, Fig. 3). The absorption of **11** in the range of 400–250 nm is consistent with the computed vertical transitions at 428, 354, 315, and 280 nm at the EOM-CCSD/aug-cc-pVDZ level of theory (Supplementary Table 2). Additionally, a very weak band in the range of 750–550 nm also belongs to **11**, as it corresponds to the computed transition at 859 nm and consequently explains its sensitivity to the red-light irradiation (830 nm). In line with the observation in the IR spectrum (Fig. 3d), the bands of **13** and **15** in the range of 230–400 vanish upon subsequent 266 nm laser irradiation. As a result, a broad band at 285 nm with onset at about 400 nm becomes discernible, and it associates with the complex mixture of the photolysis products SO_2_ (**6**), CH_3_SO_2_• (**14**), SO_3_ (**17**), and OCS (**18**). Concurrently, the characteristic absorptions at 670 and 630 nm for •NO_3_^62,63^ forming from the O_2_-oxidation of •NO in the matrix appear. Further irradiation of the matrix with UV-light (365 nm) results in the formation of unknown species with weak absorption at 500 nm in the UV–vis spectrum.

## Conclusion

In conclusion, we presented an optimized method for efficient gas-phase generation of the simplest organosulfur radical CH_3_S• (**2**) in the gas phase, opening the door to further studies on its structure and reactivity, particularly on its diverse reactions involving in Earth’s atmosphere and also the potent involvement in the astrochemistry of merthyl mercaptan (CH_3_SH) that has been recently detected in the interstellar medium (ISM)^64–66^. In addition to the first time identification of the characteristic absorption at 375 nm in the UV–vis spectrum of **2**, its photo-induced (365 nm) sulfur atom transfer SAT to molecular nitrogen has been observed in an N_2_-matrix. Furthermore, two important peroxyl radicals CH_3_SOO• (**13**) and CH_3_S(O)_2_OO• (**15**) involving in the atmospheric oxidation of dimethyl sulfide have been generated by reacting **2** with molecular oxygen and characterized using IR and UV–vis spectroscopy in cryogenic Ar-, N_2_-, and Ne-matrixes. The assignment of all the IR-active fundamental modes in the range of 4000–400 cm^−1^ for both species is supported by ^18^O- and ^13^C-isotope labeling and quantum chemical computations. The spectroscopic characterization of the sulfur-containing radical species (CH_3_SO_*x*_, *x* = 0–4) and their photochemistry in the laboratory contribute to understanding the complex mechanism for the atmospheric oxidation of dimethyl sulfide.

## Methods

### Sample preparation

*S*-Nitrosothiol (CH_3_SNO) was prepared by reacting CH_3_SH with ClNO according to the published protocol^46^. Ar (≥99.999%, Messer), N_2_ (≥99.999%, Messer), O_2_ (≥99.999%, Messer), ^15^N_2_ (98 atom %, Aldrich), ^18^O_2_ (97 atom %, Aldrich) gases were used without further purification. For the ^13^C-labeling experiments, ^13^C-MeOH (99.5%, Eurisotop) was used for the synthesis of ^13^CH_3_SH (Supplementary Methods).

### Matrix-isolation spectroscopy

Matrix IR spectra were recorded on a FT-IR spectrometer (Bruker 70 V) in a reflectance mode by using a transfer optic. A KBr beam splitter and MCT detector were used in the mid-IR region (4000–400 cm^−1^). Typically, 200 scans at resolution of 0.5 cm^−^^1^ were co-added for each spectrum. Matrix UV–vis spectra were recorded on a UV–vis spectrometer (Lambda 850+, spectral range of 800–190 nm) in a transmission mode, and a scanning speed of 2 nm s^−1^ at resolution of 1 nm was used for each spectrum. For the preparation of the matrix, the gaseous sample (CH_3_SNO) was mixed by passing a flow of N_2_ or noble gas (Ar and Ne) through a cold U-trap (−110 °C) containing ca. 20 mg of the CH_3_SNO. Then the mixture (1:1000, estimated) was passed through an aluminum oxide furnace (o.d. 2.0 mm, i.d. 1.0 mm), which can be heated over a length of ca. 30 mm by a tantalum wire (o.d. 0.4 mm, resistance 0.4 Ω). The pyrolysate was deposited (2 mmol h^−^^1^) in a high vacuum (~10^−6^ pa) onto the gold-plated copper block matrix support for IR or CaF_2_ window for UV–vis (3 K for Ne or 10 K for N_2_ and Ar) using closed-cycle helium cryostat (Sumitomo Heavy Industries, SRDK-408D2-F50H) inside the respective vacuum chambers. Temperatures at the second stage of the cold head were controlled and monitored using an East Changing T290 digital cryogenic temperature controller a Silicon Diode (DT-64). Photolysis experiments were performed using light emitting diodes (LED) (830/440 nm, 100 mW), UV flashlight (365 nm, 100 mW), and Nd^3+^:YAG laser (266 nm, MPL-F-266, 10 mW)

### Computational details

Structural optimizations and IR frequencies were computed using both DFT M06-2X/6-311++G(3df,3pd)^67^ and CCSD(T)/aug-cc-pV(X + d)Z (X = D and T)^68–70^ methods. Local minima were confirmed by vibrational frequency analysis. EOM-CCSD/aug-cc-pVDZ^71^ computations were performed for the prediction of vertical excitations. All calculations we used default threshold, for CCSD(T) calculations we use the default active space i.e., the inactive space consists of all inner-shell orbitals, and the active space of all valence orbitals which are obtained from the atomic valence orbitals (full valence active space). The DFT computations were performed using the Gaussian 09 software package^72^. The ab initio computations were performed with MOLPRO program^73^.

## Supplementary information

Supplementary Information

Description of Additional Supplementary Files

Supplementary Data 1

## Data Availability

The authors declare that all other data supporting the findings of this study are available within the paper, its Supplementary Information, and Supplementary Data 1. Additional raw data that support the findings of this study are available from the corresponding authors upon reasonable request.
