# Supplementary material for: Spectroscopic characterization of two peroxyl radicals during the O2-oxidation of the methylthio radical
Source: Commun Chem. 2022 Feb 17;5:19. doi: 10.1038/s42004-022-00637-z (PMC9814412; doi:10.1038/s42004-022-00637-z)
Supplement: Supplementary file 1 — Supplementary Information [file 42004_2022_637_MOESM1_ESM.pdf]

Supplementary Information

Spectroscopic Characterization of Two Peroxyl Radicals During the O_2–Oxidation of the Methylthio Radical

Zhuang Wu[1], Xin Shao[1], Bifeng Zhu[1], Lina Wang[1], Bo Lu[1], Tarek Trabelsi[2], Joseph S. Francisco,[2*] and Xiaoqing Zeng[1*]

[1]*Department of Chemistry, Shanghai Key Laboratory of Molecular Catalysts and Innovative Materials, Fudan University, 200433 Shanghai, China. E-mail:xqzeng@fudan.edu.cn*

[2]*Department of Earth and Environment Science, University of Pennsylvania, Philadelphia, Pennsylvania 19104-6243, USA. E-mail: frjoseph@sas.upenn.edu*

Contents:

Supplementary Methods. Synthesis of $^{13}CH_3SH$.

Supplementary Fig. 1. Pyrolysis of CH_3SNO in N_2, Ar and Ne matrices.

Supplementary Fig. 2. Matrix IR spectra showing the photochemistry of CH_3SOO• in N_2-matrix.

Supplementary Fig. 3. Matrix IR spectra showing the photochemistry of CH_3SOO• in Ne-matrix.

Supplementary Table 1. Calculated IR spectra for CH_3SOO•.

Supplementary Table 2. Calculated vertical excitation energies for CH_3SOO• and $CH_3S(O)_2OO$•.

Supplementary Table 3. Calculated IR spectra for $CH_3S(O)_2OO$•.

Supplementary Table 4. Experimental and calculated IR data for CH_3SO_2•.

Supplementary Methods. Synthesis of $^{13}CH_3SH$.

NaOH (3.0 g, 75 mmol, 5.0 equiv.) was dissolved in water (10 mL). $^{13}CH_3OH$ (1.0 g, 30 mmol, 2.0 equiv.) was added at 0 °C, and a solution of 4-methylbenzenesulfonyl chloride (2.9 g, 15 mmol, 1.0 equiv.) in tetrahydrofuran (10 mL) was added dropwise. The mixture was warmed to room temperature and stirred for 5 h. The mixture was diluted with water and extracted with ethyl acetate (3 × 20 mL). The combined organic layers were washed with saturated Na_2CO_3 solution (50 mL) and brine (50 mL) and dried over anhydrous Na_2SO_4. The solvent was evaporated under reduced pressure and the crude product was purified by flash column chromatography on silica gel (eluent: EtOAc/Petroleum ether = 1/50) to afford the ^{13}C-methyl-4-methylbenzenesulfonate (2.5 g, 13.2 mmol, 88%). The compound was prepared according to the known protocol.[1]

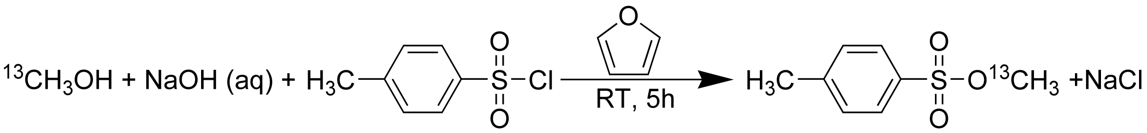

A solution of 2.5 g (13.2 mmol) of ^{13}C-methyl-4-methylbenzenesulfonate and 2.2 g (26.4 mmol) of NaSH (70%) was suspended in 8 mL of 2-methoxyethanol and refluxed (130 °C) overnight. Purification of the highly volatile product CH_3SH was performed in a vacuum line with a first U-trap at −120 °C to remove the less volatile solvent. A second cold U-trap at −196 °C was used to selectively condense CH_3SH, which was obtained in an estimated 80% yield. The compound was prepared according to the known protocol with modifications.[2]

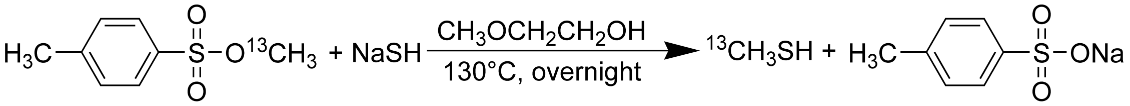

[1] Yamamoto, C., Takamatsu, K.; Hirano, K., Miura, M. *J. Org. Chem.* **81**, 7675−7684 (2016).

[2] Tuten, B. T. et al. *Angew. Chem. Int. Ed.* **58**, 5672–5676 (2019).

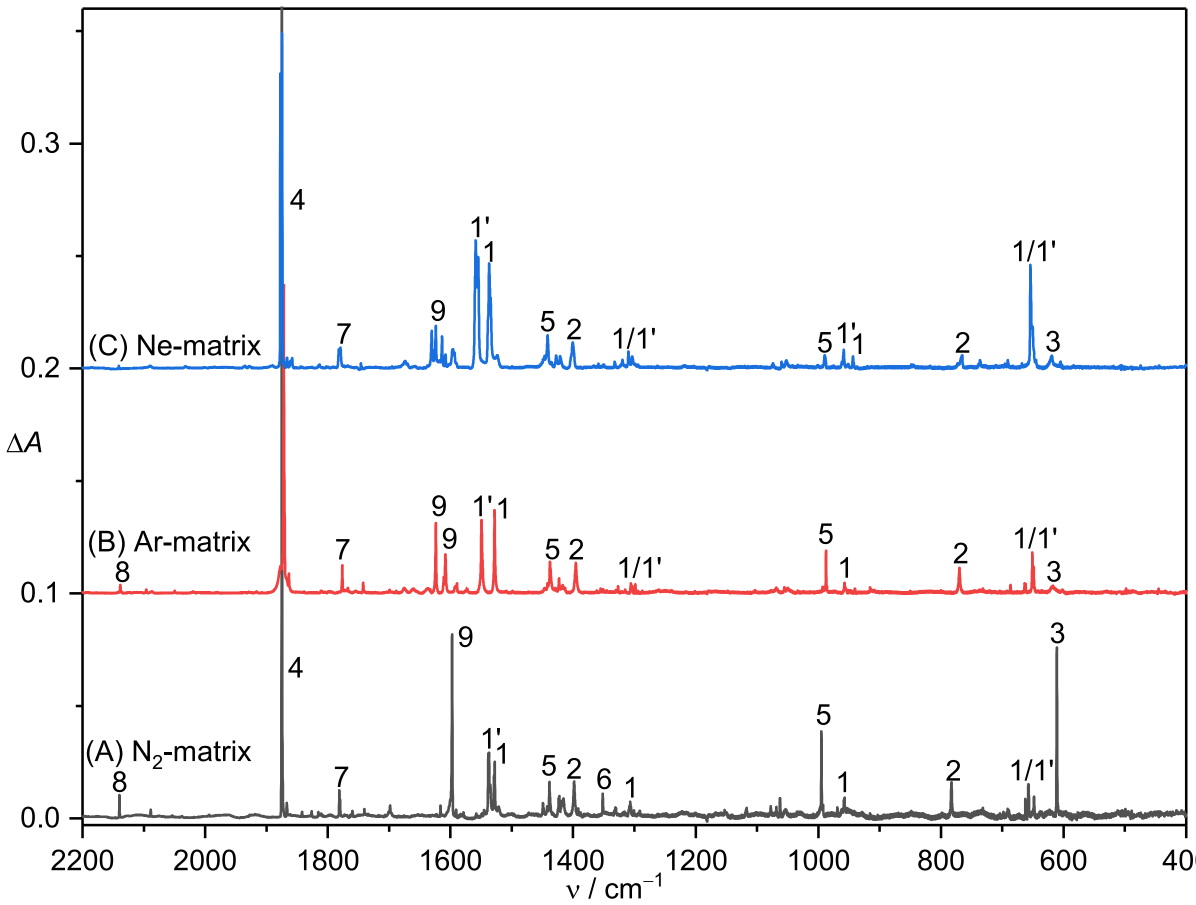

Supplementary Fig. 1. Pyrolysis of CH_3SNO in N_2, Ar and Ne matrices. IR spectra of the high-vacuum flash pyrolysis (HVFP) products of CH_3SNO (same amount of sample) in N_2-, Ar-, and Ne-matrices (A-C). The IR bands for CH_3SNO (*cis*: **1**; *trans*: **1'**), CH_3S• (**2**), CH_3• (**3**), •NO (**4**), H_2CS (**5**), SO_2 (**6**), N_2O_2 (**7**), CO (**8**), and H_2O (**9**) are labeled. As can be seen in the figure, the intensities for the IR bands of CH_3• (**3**) at about 610 cm^{-1} dramatically vary with the matrix materials. In the inert Ne- and Ar-matrices, it appears as very weak bands, however, it becomes very strong in N_2-matrix probably due to interactions with the polarizable N_2 molecules in the solid state. Changes of the IR band intensities of CH_3• (**3**) in Ar- and N_2-matrices have already been observed in a previous study, see: Milligan, D. E.; Jacox, M. E. *J. Chem. Phys.* **1967**, *47*, 5146–5156.

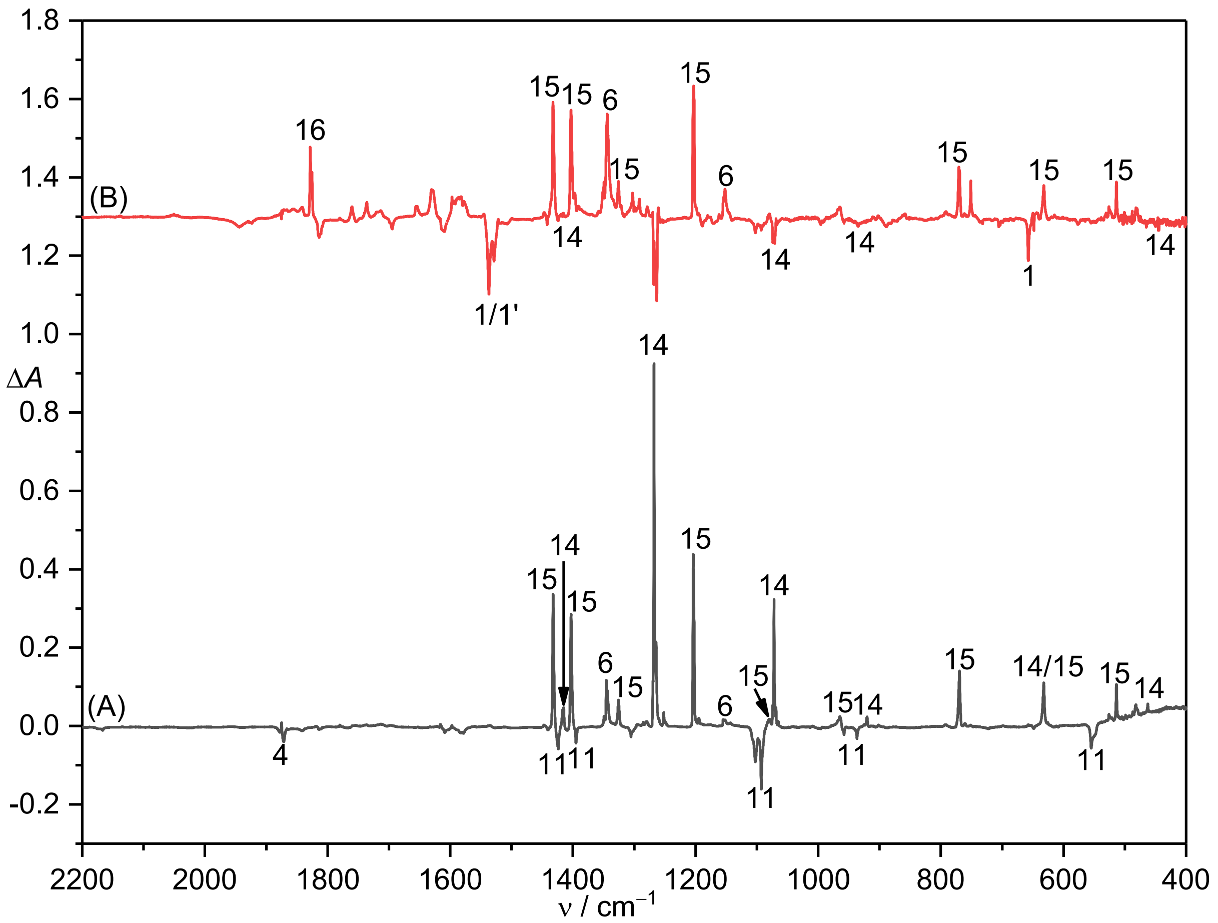

Supplementary Fig. 2. Matrix IR spectra showing the photochemistry of CH_3SOO• in N_2-matrix. (A) IR difference spectrum reflecting the change of the HVFP products of $CH_3SNO/O_2/N_2$ (1:50:1000) upon red-light irradiation (830 nm, 20 min) at 10 K. (B) IR difference spectrum reflecting the change of the HVFP products upon subsequent blue-light irradiation (440 nm, 30 min). The IR bands for CH_3SNO (*cis*: **1**; *trans*: **1'**), •NO (**4**), SO_2 (**6**), CH_3SOO• (**11**), CH_3SO_2• (**14**), $CH_3S(O)_2OO$• (**15**) and CH_3S•···•ON (**16**) are labeled.

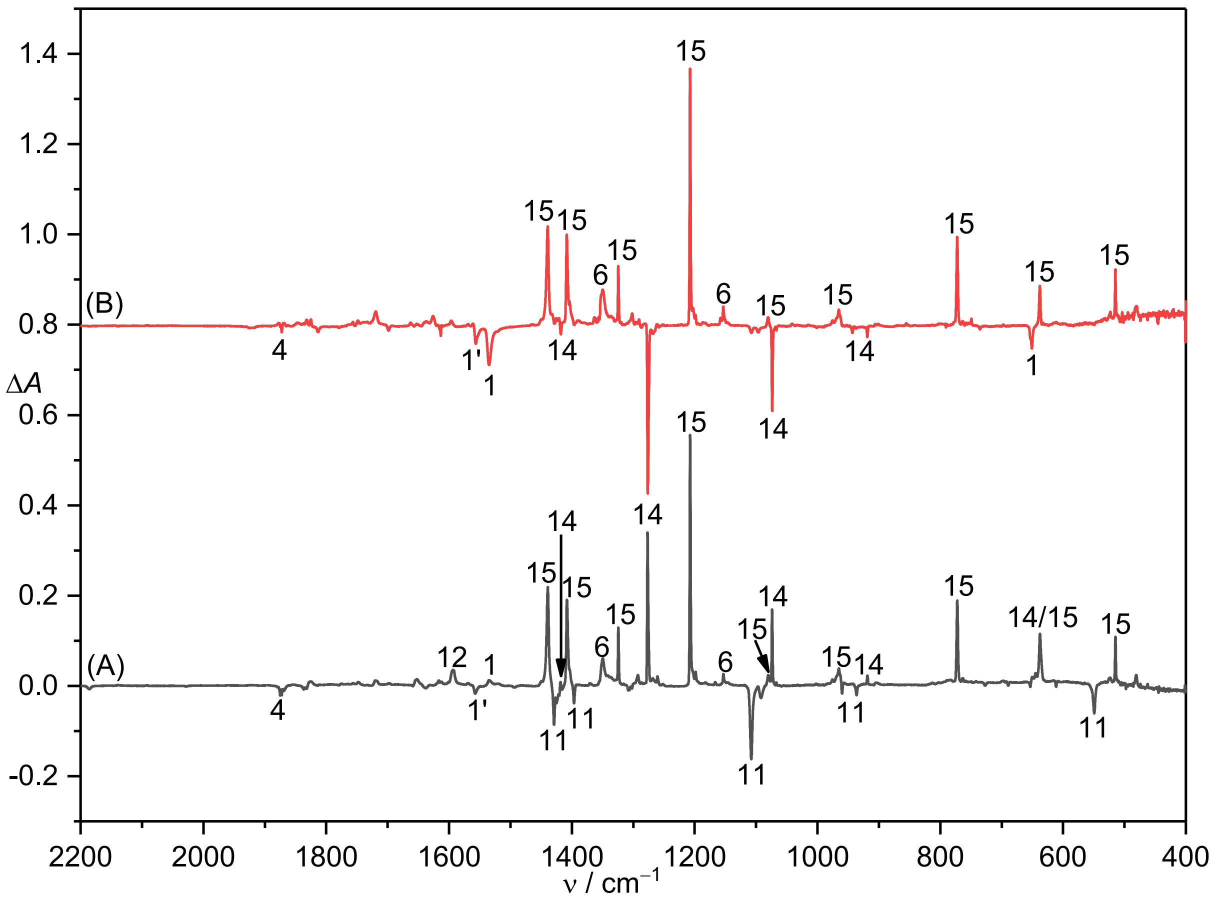

Supplementary Fig. 3. Matrix IR spectra showing the photochemistry of CH_3SOO• in Ne-matrix. (A) IR difference spectrum reflecting the change of the HVFP products of $CH_3SNO/O_2/Ne$ (1:50:1000) upon red-light irradiation (830 nm, 25 min) at 10 K. (B) IR difference spectrum reflecting the change of the HVFP products upon subsequent blue-light irradiation (440 nm, 50 min). The IR bands for CH_3SNO (*cis*: 1; *trans*: 1'), •NO (**4**), SO_2 (**6**), CH_3SOO• (**11**), NO_2• (**12**), CH_3SO_2• (**14**) and $CH_3S(O)_2OO$• (**15**) are labeled.

Supplementary Table 1. Calculated IR data for CH_3SOO•.[a]

syn-CH_3SOO•						*anti*-CH_3SOO•						
ν	ν	Δν($^{16/18}$O)		Δν($^{12/13}$C)		ν	ν	Δν($^{16/18}$O)		Δν($^{12/13}$C)		
CCSD(T)	M06-2X	CCSD(T)	M06-2X	CCSD(T)	M06-2X	CCSD(T)	M06-2X	CCSD(T)	M06-2X	CCSD(T)	M06-2X	
3158.4	3168.0 (<1)	1.8	< 0.1	13.7	12.0	3150.2	3178.7 (<1)	0.3	< 0.1	12.2	12.3	ν_1
3136.6	3147.3 (2)	1.3	< 0.1	12.1	10.7	3135.4	3162.7 (3)	0.5	< 0.1	11.5	11.3	ν_2
3044.9	3056.5 (1)	1.1	< 0.1	4.3	2.8	3045.4	3074.6 (5)	0.6	< 0.1	3.2	2.7	ν_3
1487.0	1477.0 (12)	4	< 0.1	6.5	2.6	1495.5	1489.9 (17)	1.4	< 0.1	4	2.7	ν_4
1450.1	1441.2 (9)	5.7	< 0.1	8	2.2	1465.4	1462.5 (8)	1	< 0.1	3.2	2.3	ν_5
1346.4	1344.1 (1)	– 3.1	0.9	4.1	7.3	1356.2	1360.0 (1)	-0.6	< 0.1	6.9	7.6	ν_6
1135.9	1265.7 (31)	63.8	71.5	-0.8	0.2	1066.4	1274.6 (18)	62.7	72.1	0.2	0.1	ν_7
987.1	973.9 (4)	7.1	< 0.1	12.6	5.5	990.6	986.8 (4)	1.7	0	7.3	5.5	ν_8
967.1	966.6 (5)	2.7	1.9	9	8.4	974.6	984.6 (6)	4.1	1.8	9.1	8.0	ν_9
733.5	748.1 (1)	– 0.1	0.1	14.8	15.3	730.9	743.5 (1)	-0.2	< 0.1	15.3	15.9	ν_{10}
571.1	621.4 (17)	28.2	29.0	0.8	1.0	612.0	694.1 (7)	27.9	29.0	0.4	0.1	ν_{11}
406.0	467.8 (<1)	10.7	14.4	-0.5	0.3	359.6	416.0 (2)	13.9	15.8	1.8	1.5	ν_{12}
270.1	279.0 (5)	9.3	5.2	7.8	4.1	203.9	221.3 (2)	5.5	3.7	4.3	1.7	ν_{13}
224.5	196.5 (<1)	43.0	< 0.1	42.8	< 0.1	176.8	205.5 (<1)	11.8	< 0.1	11.6	< 0.1	ν_{14}
61.8	74.4 (1)	9.8	6.7	7.5	– 0.2	70.9	94.5 (<1)	4.8	5.7	1.7	– 0.2	ν_{15}

[a] Calculated harmonic IR frequencies and isotopic shifts at the CCSD(T)/aug-cc-pV(T+d)Z and M06-2X/6-311++G(3df,3df) levels of theory.

Supplementary Table 2. Calculated of vertical excitation energies (*E*) for CH_3SOO• and $CH_3S(O)_2OO$• at the EOM- CCSD/aug-cc-pVDZ level of theory.

	syn-CH_3SOO•		*anti*-CH_3SOO•		*syn*-$CH_3S(O)_2OO$•		*anti*-$CH_3(S)O_2OO$•	
States	*E* (nm)	*f*	*E* (nm)	*f*	*E* (nm)	*f*	*E* (nm)	*f*
S1	858.6	0.0000	976.7	0.000	1334.1	0.0000	1276.6	0.0000
S2	428.2	0.0000	406.5	0.000	221.4	0.0315	224.6	0.0455
S3	354.2	0.0289	353.0	0.0383	207.6	0.0005	210.2	0.0040
S4	315.4	0.0002	310.4	0.0002	203.8	0.0116	201.3	0.0000
S5	280.8	0.0018	261.3	0.0024	199.5	0.0095	192.2	0.0011
S6	229.1	0.0004	221.6	0.0000	190.1	0.0006	186.3	0.0038
S7	221.0	0.0025	213.7	0.0007	181.8	0.0025	185.9	0.0029
S8	212.3	0.1852	210.0	0.1406	177.8	0.0021	180.9	0.0032

Supplementary Table 3. Calculated IR data for $CH_3S(O)_2OO$•.[a]

syn-$CH_3S(O)_2OO$•						*anti*-$CH_3S(O)_2OO$•						
ν	ν	Δν($^{16/18}$O)		Δν($^{12/13}$C)		ν	ν	Δν($^{16/18}$O)		Δν($^{12/13}$C)		
CCSD(T)	M06-2X	CCSD(T)	M06-2X	CCSD(T)	M06-2X	CCSD(T)	M06-2X	CCSD(T)	M06-2X	CCSD(T)	M06-2X	
3182.3	3193.8 (4)	0.2	< 0.1	12.2	12.2	3178	3186.4 (3)	0.5	< 0.1	12.5	12.1	ν_1
3167.7	3186.7 (5)	0.7	< 0.1	12.4	11.9	3167.1	3173.8 (4)	0.6	< 0.1	12.4	11.7	ν_2
3054.2	3078.6 (2)	0.9	< 0.1	3.4	2.6	3053.5	3069.7 (3)	0.6	< 0.1	3.2	2.7	ν_3
1436.0	1494.4 (191)	2.1	58.3	4.2	< 0.1	1434.7	1478.1 (188)	1.5	57.7	2.8	< 0.1	ν_4
1430.2	1459.4 (10)	1.3	1.3	2.2	2.1	1433.3	1453.0 (5)	0.8	< 0.1	3	2.0	ν_5
1367.9	1449.2 (52)	39.7	–14.9	0.7	2.0	1356.8	1442.7 (71)	34.3	0.3	0.7	1.9	ν_6
1317.2	1358.4 (34)	2.1	1.9	10.9	9.9	1322.9	1360.1 (29)	6.3	2.0	9.3	10.1	ν_7
1145.2	1255.6 (148)	44.4	49.9	0.4	0.1	1147.3	1254.2 (138)	42.8	48.6	0.5	0.2	ν_8
1061.5	1237.5 (34)	59.8	70.6	0	0	1067.9	1234.7 (25)	58.5	69.9	0.1	< 0.1	ν_9
966.8	990.6 (6)	5.5	2.8	10	8.3	965.9	978.5 (44)	3.2	3.2	8	7.7	ν_{10}
960.2	978.7 (33)	4.7	2.4	9.1	7.8	964.5	974.4 (2)	6.7	2.9	8.7	8.4	ν_{11}
757.6	802.9 (82)	8.8	8.8	9.6	9.0	760.8	806.4 (69)	7.8	8.1	9.5	10.0	ν_{12}
621.2	685.9 (64)	25.1	26.3	3.7	3.8	590.1	656.8 (70)	25.6	27.3	1.5	0.6	ν_{13}
488.9	531.4 (48)	17.9	18.5	2.3	2.3	514.3	558.4 (79)	11.1	12.8	4	5.7	ν_{14}
461.8	496.3 (49)	19.0	16.9	3.1	2.7	463.5	503.6 (27)	22.4	23.3	2.1	1.6	ν_{15}
375.5	426.4 (2)	18.2	21.3	0.9	0.2	377.8	409.9 (6)	13.4	16.7	0.7	0.4	ν_{16}
316.5	339.2 (1)	11.6	11.6	2.5	1.9	306.6	336.6 (1)	13.7	14.8	2.3	1.4	ν_{17}
283.7	304.6 (1)	11.8	11.2	3.1	2.0	293.2	307.8 (1)	11.9	11.0	2	2.3	ν_{18}
225.8	248.8 (3)	11.6	10.5	3	1.7	198.9	220.4 (2)	8.3	7.4	3.7	2.0	ν_{19}
188.2	202.0 (<1)	12.3	0.9	11.9	0.1	174	131.2 (<1)	21	0.5	22.4	< 0.1	ν_{20}
112.1	124.5 (2)	8.0	3.9	3.4	0.5	76.2	62.0 (1)	6.3	3.2	2.3	0.2	ν_{21}

[a] Calculated harmonic IR frequencies and isotopic shifts at the CCSD(T)/aug-cc-pV(D+d)Z and M06-2X/6-311++G(3df,3df) levels of theory.

Supplementary Table 4. Experimental vibrational fundamentals and calculated IR data (> 400 cm^{-1}) for CH_3SO_2•.

Obs.				Cal.		assignment[a]
Ar-matrix	Ne-matrix	N_2-matrix	$\Delta\nu(^{16/18}O)$	M06-2X	$\Delta\nu(^{16/18}O)$	
n.o.[b]	n.o.[b]	n.o.[b]	n.o.[b]	3208.8 (1)	< 0.1	ν_1, $\nu_{as}(CH_3)$
n.o.[b]	n.o.[b]	n.o.[b]	n.o.[b]	3184.7 (1)	< 0.1	ν_2, $\nu_{as}(CH_3)$
n.o.[b]	n.o.[b]	n.o.[b]	n.o.[b]	3074.2 (<1)	< 0.1	ν_3, $\nu_s(CH_3)$
1413.9	1418.6	1414.9	< 0.5	1459.2 (9)	< 0.1	ν_4, $\delta(CH_3)$
n.o.[b]	n.o.[b]	n.o.[b]	n.o.[b]	1452.1 (2)	0.2	ν_5, $\delta(CH_3)$
1274.2	1276.5	1266.8	39.0	1347.3 (166)	42.3	ν_6, $\nu_{as}(SO_2)$
n.o.[b]	n.o.[b]	n.o.[b]	n.o.[b]	1320.5 (1)	< 0.1	ν_7, $\delta(CH_3)$
1074.5	1073.8	1072.1	42.3	1146.5 (74)	49.9	ν_8, $\nu_s(SO_2)$
631.3	635.0	635.2	2.0[b]	963.5 (<1)	2.0	ν_9, $\omega(CH_3)$
915.6	918.7	920.1	0.5	942.6 (9)	1.0	ν_{10}, $\rho(CH_3)$
n.o.[b]	n.o.[b]	n.o.[b]	n.o.[b]	678.8 (14)	3.2	ν_{11}, $\nu(CS)$
460.2	462.7	462.3	16.2	474.5 (19)	17.5	ν_{12}, $\delta(SO_2)$
n.o.[b]	n.o.[b]	n.o.[b]	n.o.[b]	393.8 (25)	9.9	
n.o.[b]	n.o.[b]	n.o.[b]	n.o.[b]	309.7 (<1)	8.0	
n.o.[b]	n.o.[b]	n.o.[b]	n.o.[b]	175.8 (<1)	0.6	

[a]Tentative assignment of the vibration modes based on the vibrational displacement vectors.
[b]Not observed due to overlap or low intensity.
