# Supplementary material for: Spectroscopic characterization of two peroxyl radicals during the O2-oxidation of the methylthio radical
Source: Commun Chem. 2022 Feb 17;5:19. doi: 10.1038/s42004-022-00637-z (PMC9814412; doi:10.1038/s42004-022-00637-z)
Supplement: Supplementary file 2 — Description of Additional Supplementary Files [file 42004_2022_637_MOESM2_ESM.pdf]

Description of Additional Supplementary Files

File Name: Supplementary Data 1

Description: Calculated structures (selected bonds in Angstroms and angles in degree), energies (in Hartree), atomic coordinates (in Angstroms), and IR data for all optimized species
