# Supplementary material for: Spectroscopic characterization of two peroxyl radicals during the O2-oxidation of the methylthio radical
Source: Commun Chem. 2022 Feb 17;5:19. doi: 10.1038/s42004-022-00637-z (PMC9814412; doi:10.1038/s42004-022-00637-z)
Supplement: Supplementary file 3 — Supplementary Data 1 [file 42004_2022_637_MOESM3_ESM.pdf]

Calculated structures (selected bonds in Angstroms and angles in degree), energies (in Hartree), atomic coordinates (in Angstroms), and IR data for all optimized species.

***syn*-CH_3SOO•**
M06-2X/6-311++G(3df,3pd)

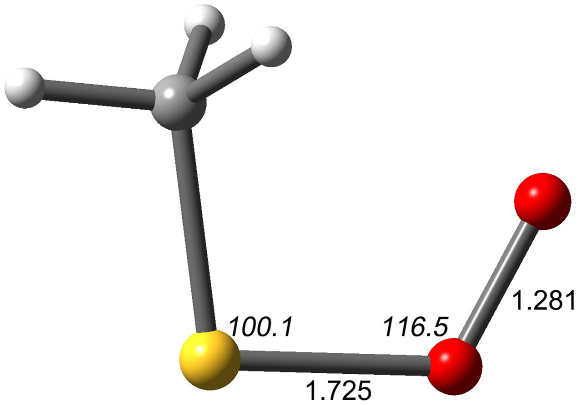

Molecular Symmetry: C_s
Temperature= 298.150000 Pressure= 1.000000

Item	Value	Threshold	Converged?
Maximum Force	0.000036	0.000450	YES
RMS Force	0.000019	0.000300	YES
Maximum Displacement	0.001222	0.001800	YES
RMS Displacement	0.000674	0.001200	YES

Zero-point correction=	0.043805 (Hartree/Particle)
Thermal correction to Energy=	0.049044
Thermal correction to Enthalpy=	0.049988
Thermal correction to Gibbs Free Energy=	0.014538
Sum of electronic and zero-point Energies=	-588.333882
Sum of electronic and thermal Energies=	-588.328642
Sum of electronic and thermal Enthalpies=	-588.327698
Sum of electronic and thermal Free Energies=	-588.363149

Cartesian coordinates (x, y, z, in Å)

C	1.15044200	0.98561700	-0.00052900
H	2.23861100	0.92333300	-0.00329500
H	0.80764600	1.49932500	-0.89321500
H	0.81212800	1.49971400	0.89358500
S	0.62709700	-0.71405500	0.00062200
O	-1.08540900	-0.50449600	-0.00156100
O	-1.51391400	0.70309600	0.00107900

Vibrations frequencies (cm^{-1}) and IR intensities (km mol^{-1})

3168.0738	0.0137
3147.3188	2.1528
3056.5029	1.8539
1477.0299	12.4109
1441.2311	9.4988
1344.1566	0.9081
1265.7504	31.5800

973.9881	4.1147
966.6211	5.7849
748.1937	0.9667
621.4127	17.6845
467.8609	0.5476
279.0409	5.3369
196.5143	0.0538
74.4638	1.0774

***anti*-CH_3SOO•**
M06-2X/6-311++G(3df,3pd)

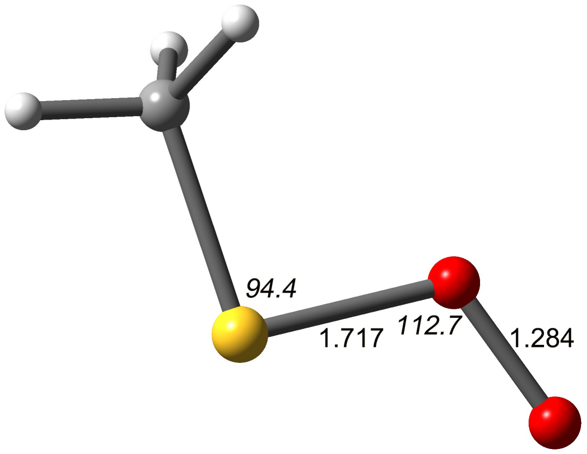

Molecular Symmetry: C_s
Temperature= 298.150000 Pressure= 1.000000

Item	Value	Threshold	Converged?
Maximum Force	0.000198	0.000450	YES
RMS Force	0.000063	0.000300	YES
Maximum Displacement	0.001198	0.001800	YES
RMS Displacement	0.000458	0.001200	YES

Zero-point correction=	0.044082 (Hartree/Particle)
Thermal correction to Energy=	0.049355
Thermal correction to Enthalpy=	0.050300
Thermal correction to Gibbs Free Energy=	0.014940
Sum of electronic and zero-point Energies=	-588.331930
Sum of electronic and thermal Energies=	-588.326657
Sum of electronic and thermal Enthalpies=	-588.325713
Sum of electronic and thermal Free Energies=	-588.361072

Cartesian coordinates (x, y, z, in Å)

C	1.74353800	0.51331500	0.00002000
H	2.64368200	-0.09976700	0.00011700
H	1.72866600	1.12595500	-0.89646300
H	1.72854500	1.12605200	0.89643400
S	0.38711000	-0.64803800	-0.00000800
O	-0.82810300	0.56509100	-0.00004700
O	-2.01638200	0.07696800	0.00003800

Vibrations frequencies (cm^{-1}) and IR intensities (km mol^{-1})

3178.7040	0.4147
3162.7892	3.6704
3074.6479	5.1954
1489.9124	17.4001
1462.4959	8.5837
1360.0162	0.5789
1274.6742	18.3002
986.8847	4.3826
984.5971	6.4555
743.5751	0.8773
694.1431	7.6233
415.9961	2.4840
221.3256	2.0808
205.5355	0.2579
94.5657	0.0162

***syn*-$CH_3S(O)_2OO$•**
M06-2X/6-311++G(3df,3pd)

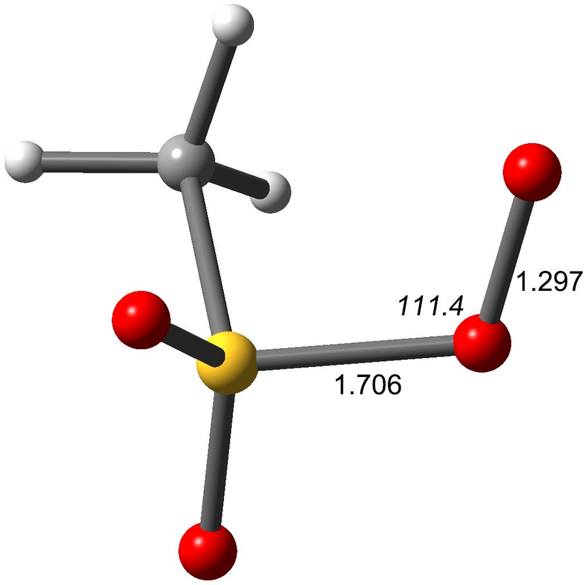

Molecular Symmetry: C_1
Temperature= 298.150000 Pressure= 1.000000

Item	Value	Threshold	Converged?
Maximum Force	0.000082	0.000450	YES
RMS Force	0.000033	0.000300	YES
Maximum Displacement	0.001791	0.001800	YES
RMS Displacement	0.000810	0.001200	YES

Zero-point correction=	0.054324 (Hartree/Particle)
Thermal correction to Energy=	0.060720
Thermal correction to Enthalpy=	0.061664
Thermal correction to Gibbs Free Energy=	0.023411
Sum of electronic and zero-point Energies=	-738.773041
Sum of electronic and thermal Energies=	-738.766645
Sum of electronic and thermal Enthalpies=	-738.765701
Sum of electronic and thermal Free Energies=	-738.803955

Cartesian coordinates (x, y, z, in Å)

C	-0.43033	1.61771	-0.11664
H	0.41181	2.00658	0.44681
H	-0.35928	1.84607	-1.1751
H	-1.37188	1.96936	0.29762
S	-0.44705	-0.13068	0.06793
O	-0.37513	-0.48072	1.43726
O	-1.34789	-0.73816	-0.83997
O	1.06566	-0.5002	-0.62879
O	2.03913	0.0394	0.03697

Vibrations frequencies (cm^{-1}) and IR intensities ($km\ mol^{-1}$)

3193.8292	4.3568
3186.7345	5.3121
3078.6743	2.5662

1494.3365	191.7686
1459.4316	10.7047
1449.2400	52.3835
1358.4435	34.3694
1255.6228	148.4894
1237.5232	34.1218
990.6764	6.1724
978.7312	33.1277
802.9306	82.6574
685.9681	64.6401
531.4222	48.5270
496.3804	49.8058
426.4747	2.8546
339.2395	0.7544
304.6743	0.5677
248.8060	3.7822
202.0011	0.1618
124.5011	2.3735

***anti*-$CH_3S(O)_2OO$•**
M06-2X/6-311++G(3df,3pd)

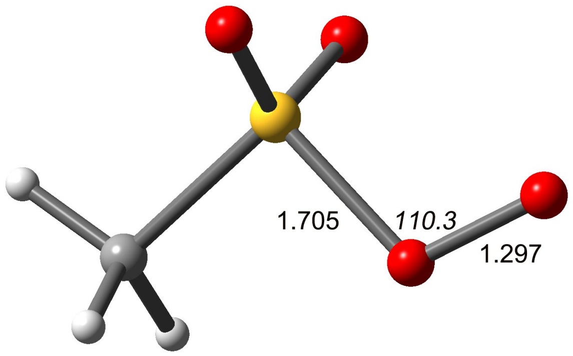

Molecular Symmetry: C_s
Temperature= 298.150000 Pressure= 1.000000

Item	Value	Threshold	Converged?
Maximum Force	0.000133	0.000450	YES
RMS Force	0.000045	0.000300	YES
Maximum Displacement	0.000576	0.001800	YES
RMS Displacement	0.000267	0.001200	YES

Zero-point correction=	0.053764 (Hartree/Particle)
Thermal correction to Energy=	0.060448
Thermal correction to Enthalpy=	0.061392
Thermal correction to Gibbs Free Energy=	0.021981
Sum of electronic and zero-point Energies=	-738.770081
Sum of electronic and thermal Energies=	-738.763397
Sum of electronic and thermal Enthalpies=	-738.762453
Sum of electronic and thermal Free Energies=	-738.801863

Cartesian coordinates (x, y, z, in Å)

C	1.55120900	1.03479600	0.00034400

H	1.43047000	1.62734100	-0.90084200
H	1.42993700	1.62746700	0.90137500
H	2.50886400	0.52044300	0.00065900
S	0.33059300	-0.22920500	0.00009000
O	0.30290500	-0.91486800	-1.23912400
O	0.30200200	-0.91449200	1.23948900
O	-0.99097400	0.84869200	-0.00057300
O	-2.10968400	0.19107500	-0.00037900

Vibrations frequencies (cm^{-1}) and IR intensities ($km\ mol^{-1}$)

3186.4651	3.2778
3173.8940	4.3411
3069.7107	3.6005
1478.1588	188.1134
1453.0649	5.9662
1442.7715	71.8144
1360.1084	29.0009
1254.2543	138.2762
1234.7906	25.8383
978.5833	44.0295
974.4185	2.7472
806.4427	69.2706
656.8605	70.0793
558.4558	79.6827
503.6771	27.2627
409.9115	6.8019
336.6542	1.5635
307.8732	0.8544
220.4174	2.5360
131.2781	0.0515
62.0202	0.1004

***anti*-CH_3SOO•**

UCCSD(T)/aug-cc-pV(T+d)Z

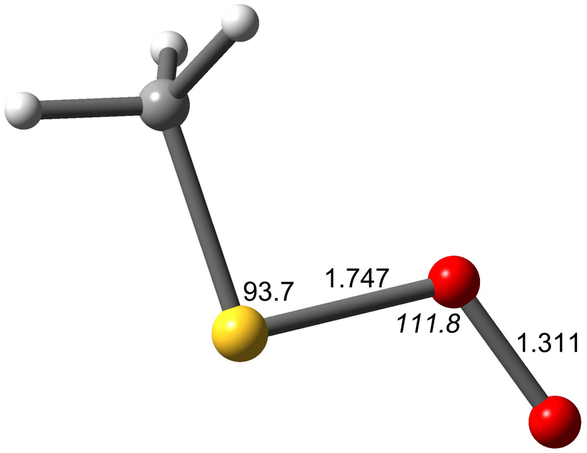

Molecular Symmetry: C_s

Temperature= 298.150000 Pressure= 1.000000

ENERGY=-587.68919536

Cartesian coordinates (x, y, z, in Å)

C	1.7520537719	0.5120856347	0.0000257719
H	2.6591652704	-0.0968776705	0.0000844713
H	1.7299121983	1.1264187771	-0.8992737565
H	1.7298546982	1.1265074002	0.8992635102
S	0.3932764202	-0.6627492178	0.0000420541
O	-0.8340976038	0.5814157589	-0.0000489497
O	-2.0431087553	0.0727753174	-0.0000021013

Vibrations frequencies (cm^{-1})
3150.2
3135.4
3045.4
1495.5
1465.4
1356.2
1066.4
990.6
974.6
730.9
612.0
359.6
203.9
176.8
70.9

***syn*-CH_3SOO•**
UCCSD(T)/aug-cc-pV(T+d)Z

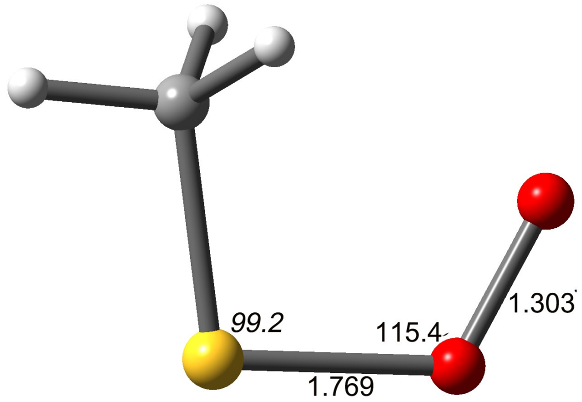

Molecular Symmetry: C_s
Temperature= 298.150000 Pressure= 1.000000
ENERGY=-587.69073534

Cartesian coordinates (x, y, z, in Å)

C	1.1551402813	0.9848822162	-0.0010125033
H	2.2478005922	0.9381749276	-0.0019098156
H	0.8021332709	1.4931199276	-0.8967764553

H	0.8035831116	1.4928252505	0.8954728113
S	0.6502746596	-0.7311388624	-0.0009390658
O	-1.1058242956	-0.5112916676	-0.0001602578
O	-1.5165066202	0.7259622081	0.0020112865

Vibrations frequencies (cm^{-1})
3158.4
3136.6
3044.9
1487.0
1450.1
1346.4
1135.9
987.1
967.1
733.5
571.1
406.0
270.1
224.5
61.8

***syn*-$CH_3S(O)_2OO$•**
UCCSD(T)/aug-cc-pV(D+d)Z

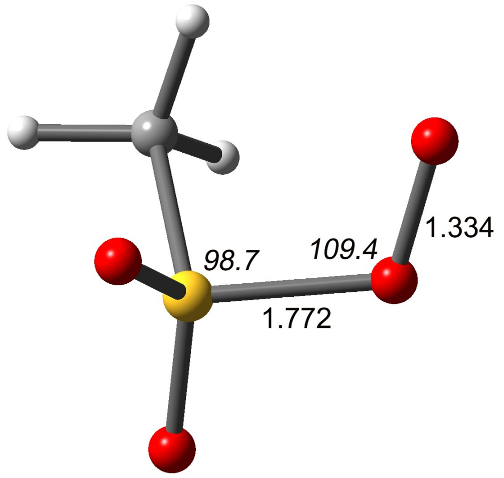

Molecular Symmetry: C_1
Temperature= 298.150000 Pressure= 1.000000
ENERGY=-737.60596305

Cartesian coordinates (x, y, z, in Å)

C	-0.4375576462	1.6312782649	-0.1170304720
H	0.4254911452	2.0139826762	0.4484391571
H	-0.3670022081	1.8549595347	-1.1924440111
H	-1.3878715287	1.9942043762	0.3076967785
S	-0.4640742174	-0.1407950086	0.0730822317
O	-0.3795678358	-0.4921821648	1.4769002797

O	-1.3845350128	-0.7586531005	-0.8635196408
O	1.1030027880	-0.5140204944	-0.6651299726
O	2.0771525157	0.0405939165	0.0580866496

Vibrations frequencies (cm^{-1})
3182.3
3167.7
3054.2
1436.0
1430.2
1367.9
1317.2
1145.2
1061.5
966.8
960.2
757.6
621.2
488.9
461.8
375.5
316.5
283.7
225.8
188.2
112.1

***anti*-$CH_3S(O)_2OO$•**
UCCSD(T)/aug-cc-pV(D+d)Z

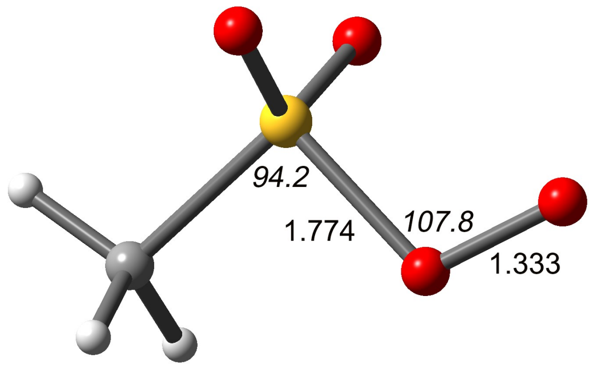

Molecular Symmetry: C_s
Temperature= 298.150000 Pressure= 1.000000
ENERGY=-737.60252101

Cartesian coordinates (x, y, z, in Å)

C	1.5703953498	1.0404065374	0.0003486749
H	1.4410509606	1.6399811067	-0.9139375106
H	1.4406266009	1.6401435947	0.9144674344
H	2.5434899203	0.5225151794	0.0006165201
S	0.3358901620	-0.2428590431	0.0001482568
O	0.3048233058	-0.9369771072	-1.2741609658

O	0.3040186318	-0.9366371410	1.2746214825
O	-1.0297932344	0.8899306154	-0.0004354231
O	-2.1551796969	0.1747452578	-0.0006294692

Vibrations frequencies (cm^{-1})
3178
3167.1
3053.5
1434.7
1433.3
1356.8
1322.9
1147.3
1067.9
965.9
964.5
760.8
590.1
514.3
463.5
377.8
306.6
293.2
198.9
174
76.2
